# The Current Landscape of Phage–Antibiotic Synergistic (PAS) Interactions

**DOI:** 10.3390/antibiotics14060545

**Published:** 2025-05-27

**Authors:** Brittany S. I. Supina, Jonathan J. Dennis

**Affiliations:** Department of Biological Sciences, University of Alberta, Edmonton, AB T6G 2E9, Canada; supina@ualberta.ca

**Keywords:** bacteriophages, antibiotics, phage therapy, synergy, antagonism, phages, novel therapeutics

## Abstract

**Background:** In response to the urgent need for new antibiotics targeting high-priority MDR pathogens, bacteriophages (phages) have emerged as promising non-traditional antimicrobial agents. Phages are viruses that infect bacteria and induce cell lysis through mechanisms distinct from those of antibiotics, making them largely unaffected by most antibiotic resistance mechanisms. Importantly, phages have been shown to work cooperatively with an array of clinically useful antibiotics, and phage–antibiotic synergy (PAS) represents a sophisticated strategy that may improve treatment outcomes. However, the interactions between phages and antibiotics are diverse, ranging from synergistic to antagonistic, and understanding the mechanisms underlying these interactions is crucial for developing effective PAS treatments. In this review, we summarize the potential evolutionary and molecular mechanisms that drive PAS and the current landscape of phage–antibiotic interactions. **Conclusions**: Towards the development of robust PAS strategies, we review in vitro methods for assessing PAS and considerations for choosing and employing candidate phage–antibiotic combinations.

## 1. Introduction

### 1.1. Antimicrobial Resistance

Antimicrobial-resistant (AMR) bacterial pathogens pose a veritable threat to human health that requires urgent care. In 2021 alone, a staggering 4.71 million deaths were associated with AMR and new forecasts predict that this number will climb to 8.22 million deaths per year by 2050 [1]. While these forecasts present a grim future, researchers suggest that 11.1 million deaths in the next 25 years could be prevented through the development of new antimicrobials targeting Gram-negative bacteria [1]. This pressing need for new antimicrobials targeting Gram negatives has also been advocated by the World Health Organization, who identified research and development of compounds with novel mechanisms of action and compounds that target antibiotic resistance mechanisms as important areas of innovation in their 2024 Bacterial Priority Pathogens List [2]. However, bioprospecting for novel antibiotics is challenging; it can require the lengthy culturing of rare or fastidious organisms [3,4], and despite these efforts, new classes of antibiotics are rarely found. Indeed, reports indicate that out of the twelve new antibiotics brought to market between 2017 and 2022, only one represented a novel class [5]. More recently, researchers have discovered a novel lasso peptide with broad activity against priority pathogens such as *Acinetobacter baumanii* [3]. But, despite these recent successes, AMR infections continue to rise and the development of mechanistically unique antimicrobials targeting priority pathogens remains urgent [5].

For Gram-negative pathogens, which represent the majority of the high-priority pathogens identified by the WHO [2], antibiotic resistance often occurs at the level of drug uptake [6]. Although several variables including compound size, structure, and hydrophobicity impact the uptake of antibiotics, many antibiotics enter Gram-negative bacterial cells through outer membrane channels or porins [7,8], which can be downregulated or altered to prevent drug influx [6,9,10]. Other drugs that gain access to cells through porin-independent self-promoted uptake mechanisms may be blocked by lipopolysaccharide (LPS) modifications that interfere with charge-specific drug–membrane interactions [6]. Additionally, the production of capsule polysaccharides may protect some pathogens against membrane-disrupting compounds, though some debate exists [11,12]. Whereas many of these mechanisms are exclusive to Gram-negative bacteria, some Gram-positive bacteria also may alter their cytoplasmic membranes to prevent drug influx [13]. Therefore, decreased cell permeability provides a frontline defense against antibiotics across diverse pathogen groups.

Decreased cell permeability provides the highest levels of antibiotic resistance when coupled with the extrusion of antimicrobials by multidrug efflux pumps. These pumps, which comprise six distinct families, use ATP or energy produced across ion gradients to expel a range of toxic substrates out of the cytoplasmic space [14]. Efflux plays a key role in the collective antimicrobial response by reducing drug accumulation, but some antibiotics may still gain access to their bacterial targets. In this case, intracellular mechanisms may counter their activity [6]. For instance, drug targets may be modified to prevent drug binding [15], antibiotics may be directly inactivated or altered by protective enzymes [16], or the drug-mediated deactivation of bacterial machinery may be overcome through functional redundancies [6]. For an extensive review of current AMR mechanisms, refer to work by Darby et al. [6]. Given that drug resistance consistently involves these primary mechanisms, the application of compounds that increase cell permeability, decrease efflux, or inhibit drug-inactivating enzymes has been proposed as an intriguing strategy to improve the activity of existing antibiotics [6].

### 1.2. Bacteriophage Therapy

Bacteriophages, or phages, are viruses that specifically infect bacteria that were first used by Félix d’Hérelle in the early 1900s to treat children suffering from bacterial dysentery [17]. These initial successes preceded a period of great interest in phage therapy in France and across the globe [17]. Although phage-based therapies were soon after abandoned by Western nations in favor of traditional antibiotics, the current antibiotic resistance crisis has renewed global interest in phage research [17]. Recently, several phages and phage-derived products have been investigated in clinical trials for the treatment of priority pathogens including *Staphylococcus aureus* and *Pseudomonas aeruginosa* [5]. The growing popularity of phage-based strategies relates to their unique mechanism of action. Unlike antibiotics, phage activity is highly specific and requires the irreversible binding of phage-encoded receptor-binding proteins (RBPs) to unique, specific bacterial structures, (i.e., receptors) [18]. These receptors are diverse, but are typically LPS, flagella, type 4 pili (T4P), capsule, or outer membrane proteins for Gram-negative phages, and flagella, teichoic acid, or peptidoglycan for phages infecting Gram-positive pathogens (reviewed in [18]). Conformational changes to the phage particle upon receptor binding initiate the translocation of phage genomic material into the host cell [18].

For obligately lytic (or virulent) phages, genome injection is followed by DNA replication and the expression of phage-encoded proteins, which assemble into phage particle progeny that are eventually released from the cell through the action of phage-encoded lysis proteins [19]. This process usually requires commandeered bacterial replication machinery such as RNA polymerase and ribosomes [20,21]. However, certain groups of phages produce their own RNA polymerases, which may reduce phage reliance on bacterial transcriptional machinery for the production of early phage proteins or during all stages of phage gene expression [22,23,24]. For lysogenic (or temperate) phages, genome injection may initiate lytic growth, or may usher in a state of cellular dormancy where the phage genome replicates along with the bacterial genome as an integrated chromosomal element or phage-plasmid without producing progeny or causing cell lysis [19]. In this state, some temperate phages may increase bacterial virulence through a process called lysogenic conversion [25], or may protect the newly formed lysogen from secondary infection by other phages through sundry exclusionary mechanisms [26,27,28,29,30]. Also, phage genome integration postpones phage-driven bacterial lysis indefinitely, reducing the antibacterial potential of phage therapy [31]. Although the integrative nature of temperate phages poses therapeutic challenges, they are abundant in nature [32] and show potential as therapeutic agents for clinical pathogens [33], making them an unexploited reserve of antimicrobial agents. Nonetheless, additional research is required to develop temperate-phage strategies that match the safety and efficacy of lytic phages [31].

Although the unique mechanism of action of bacteriophages makes them attractive for fighting MDR pathogens, bacteria have evolved an arsenal of antiphage defense systems that target invading phage components or processes [34,35]. Also, phage resistance can arise rapidly due to mutational or regulatory processes that reduce receptor availability [36,37]. While phage resistance poses a challenge for phage therapy, phage cocktails composed of multiple phages targeting different receptors [38] or different components of the same receptor [39] may help to reduce receptor-mediated phage resistance, and have been the focus of extensive recent work [40]. Additionally, the mutation of receptors with important cell functions may be exploited to steer bacterial populations toward states of reduced virulence [36], reduced fitness [41], or increased antimicrobial susceptibility [42,43,44]. These new evolutionary frameworks have been identified as crucial areas of study in the modern era of phage therapy [37].

### 1.3. Phage–Antibiotic Synergy (PAS)

For MDR pathogens, bacteriophages may be especially useful in combination with antibiotics [45]. During routine disk diffusion antibiotic susceptibility testing of a urine sample, Comeau et al. [46] observed phage plaques growing on a lawn of uropathogenic *Escherichia coli*. The researchers noted that the plaques were discernably larger in areas containing sub-inhibitory amounts of aztreonam and cefixime, indicating a synergistic interaction between these β-lactam antibiotics and bacteriophages within the sample. In that work, the phenomenon of increased phage production due to antibiotic action at subinhibitory levels was coined phage–antibiotic synergy (PAS) [46]. Since then, PAS has become a crucial area of phage research that has produced several clinical successes [47,48]. Currently, the assays, parameters, and models used to define interactions between antimicrobials vary; however, in general, synergy refers to antimicrobial effects that surpass the expected combined (i.e., additive) effects of the two antimicrobial agents, whereas antagonism results when the observed combined effect is less than seen for individual antimicrobial agents [33,49].

Although the early descriptions of PAS centered on the direct modulation of phage growth by antibiotics [46], growing interest in the complex evolutionary dynamics of phage–host interactions has expanded the mechanisms of PAS to consider new modes of synergy [45]. Despite these advances, knowledge gaps surrounding the mechanisms of synergy and antagonism exist [50], and largely prevent the pattern-based prediction of effective phage–antibiotic pairs without prior experimental screening. In this review, we summarize established mechanisms of PAS, prioritizing recent advances in PAS that expand upon previous findings or reveal new mechanisms of action. Furthermore, we review the known mechanisms of antagonism and highlight inconsistencies that prevent the widespread generalization of antagonism across phage–antibiotic pairs. Through this mechanism-centered lens, we review considerations toward the design, testing, and implementation of phage–antibiotic treatments.

## 2. Potential Mechanisms of PAS

**Figure 1:**
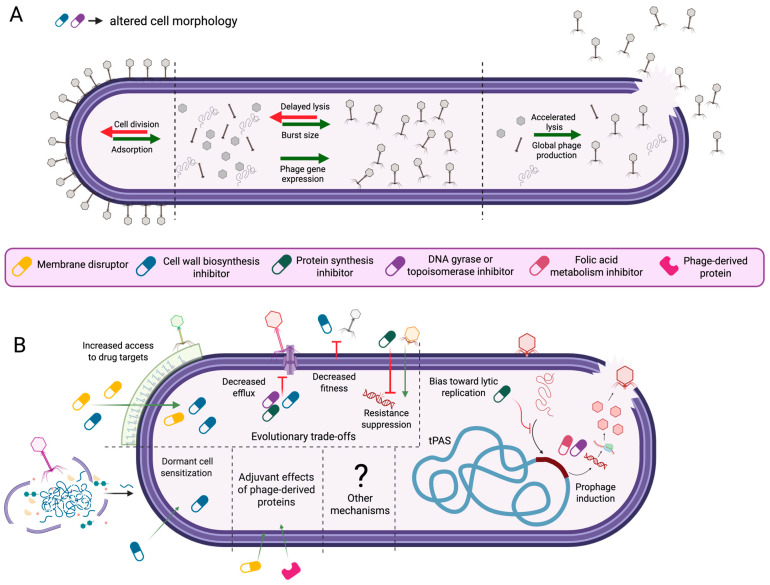
The potential mechanisms of PAS. (**A**) Traditional modes of PAS associated with cell morphology changes and increased phage replication. Possible mechanisms include increased phage adsorption to elongated cells [53,54], increased phage production per cell through increased burst size via delayed lysis [55], or increased phage gene expression [56] and increased global phage production through accelerated lysis [46,54]. (**B**) Temperate phage–antibiotic synergy (tPAS), evolutionary trade-offs, and other potential mechanisms. Antibiotics may increase the lytic activity of temperate bacteriophages by modulating the lysis–lysogeny switch and preventing lysogeny during the early stages of infection [57], or by inducing newly integrated prophages from the host genome [58]. Furthermore, bacteriophages recognizing antibiotic resistance-associated structures such as lipopolysaccharide, capsule, or efflux pumps may exert selective pressure on pathogens to mutate or alter these structures, which in turn increases antibiotic sensitivity [44,59,60]. Simultaneous evolutionary pressure by phages and antibiotics may result in other fitness trade-offs or may reduce the emergence of phage-resistant bacteria [56,61]. Moreover, phages capable of replicating inside deep dormant bacteria may aid in sensitizing these antibiotic-tolerant microbes to antibiotic action [62]. As an alternative to whole phage particles, purified or engineered phage lysins and phage-derived depolymerases may potentiate the activity of certain antibiotics by increasing access of antibiotics to their cellular targets [63,64]. As indicated in the legend, examples of the general antibiotic groups or phage-derived proteins that may engage in each type of interaction in specific phage-host systems are shown. Created with BioRender.com.

Early PAS studies established cell filamentation as a crucial aspect of synergy [46,51], and defined PAS based on the potential of certain antibiotics to increase phage growth [46]. While this traditional mechanism of PAS remains relevant, it is widely accepted that the antibacterial activity of phage–antibiotic combinations extends beyond this original definition [45,52]. Here, we review the current state of research surrounding PAS mechanisms (Figure 1).

### 2.1. Changes to Cell Morphology

#### 2.1.1. Antibiotic-Induced Changes to Host Cell Morphology and Phage Infection Dynamics

Cell filamentation is a protective morphological state that improves cell survival under a range of environmental stressors and is linked to arrested cell division [65]. Filamentation is well known to occur through activation of the conserved bacterial SOS stress response [65], though other less-characterized pathways of filamentation induction also exist [66]. Normally, SOS expression is repressed by the autoregulatory repressor protein, LexA (reviewed in [65]). Under DNA-damaging conditions, recognition of ssDNA activates the repair recombinase, RecA, to promote the self-cleavage of LexA [65]. This self-cleavage causes the de-repression of bacterial SOS genes, allowing for the expression of bacterial DNA repair systems. Also, SOS de-repression results in the expression of SulA, which inhibits the assembly of the essential cell division protein FtsZ into its active structure [67]. Consequently, the growing cell fails to divide and forms an elongated, multinucleate filament.

Both DNA replication-inhibiting fluoroquinolones [68] and cell wall biosynthesis-inhibiting β-lactams have been shown to induce the SOS response in *S. aureus* and *E.coli* [69,70], but the mechanisms of induction differ slightly across antibiotics. While fluoroquinolones directly cause double-stranded DNA breaks which induce the SOS response [71,72], β-lactams exert antimicrobial effects by inhibiting cell wall biosynthesis penicillin-binding proteins (PBP’s) [73]. Several PBPs including PBP1 in *Bacillus subtilis* and PBP3 in *E coli* also play a role in cell division [73], and the inactivation of PBP3 in *E. coli* has been shown to induce the SOS response [70]. Notably, trimethoprim, which causes DNA damage through the inhibition of dihydrofolate reductase, may also induce filamentation in some bacteria [74].

In their foundational work, Comeau et al. directly connected the morphological state of filamentation to improved phage activity, showing that a constitutively filamented strain of *E. coli* (*ftsZ-*) produced larger plaques even in the absence of an antibiotic [46]. Notably, in that work, this effect did not depend upon the activation of the SOS response, as mutations in *recA* and *sulA* did not abolish synergistic effects [46]. Nonetheless, filamentation was identified as a crucial factor in PAS [46]. Since then, several studies have reiterated this important connection, finding that synergy in certain phage–host systems could occur with filamentation or elongation-inducing antibiotics, but not with antibiotics that do not elicit morphological changes [51,53]. Mechanistically, antibiotic-induced filamentation may increase phage activity by improving host recognition or by modulating the rate and kinetics of phage replication [53]. But, as described in this section, the precise impacts vary across systems.

#### 2.1.2. Morphology-Driven Modulation of Phage–Host Interactions

Given their increased surface area, filamented cells seem to provide more opportunity for phage–bacteria interactions to occur in space; yet, direct proof of this mechanism is difficult to establish, and differences across studies suggest it may be system-dependent [53,54,55,75]. For instance, Kamal et al. [75], found that subinhibitory concentrations of ciprofloxacin, tetracycline, or meropenem caused cell filamentation, elongation, or clumping, respectively, in the *Burkholderia cenocepacia* strains K56-2 and C6433. In plaque-size assays, synergy between phages KS14 and KS12 and all three antibiotics was observed, suggesting that cell morphological changes beyond filamentation are congruent with increased phage activity. Researchers speculated that cell filamentation and elongation may enhance the likelihood of phage–receptor interactions, whereas cell clumping may facilitate improved access of phages to adjacent cells [75]. Further experimental support for this hypothesis was shown by Davis et al., during the investigation of LPS-binding phage E79 and *Pseudomonas aeruginosa* PAO1 [54]. Transmission electron micrographs showed an increase in cell-associated phages from 2 ± 1 phages per cell to 5 ± 2 phages/cell after PAO1 was grown in filamentation-inducing concentrations of aztreonam lysine [54], supporting increased adsorption to filamented cells. More recently, the role of cell elongation in PAS has been corroborated for *Escherichia coli* phages HK620 and T5 [53]. Using epifluorescence microscopy to visualize phage–host interactions, researchers found that the percentage of cells with at least one adsorbed HK620 phage increased from 34.2% in untreated culture to 48.4% or 63% when cultures were challenged with subinhibitory ciprofloxacin or cephalexin. Furthermore, researchers observed morphological heterogeneity within antibiotic-treated cultures and found that elongated cells were associated with phage particles 58.4% or 67.2% of the time in ciprofloxacin- or cephalexin-treated cultures, whereas only 37.7% or 46.5% of morphologically normal cells were associated with one or more phage particles under each antibiotic condition, respectively. Subsequent phage per surface area calculations suggested that the increased cell size of filamented cells mediated increased adsorption.

In contrast to these studies and others [56] that suggest cell filamentation biases toward a successful receptor-mediated phage adsorption, some researchers have reported that antibiotic-induced filamentation does not enhance phage binding [55]. Notably, Kim et al. observed a transient initial decrease in T4 phage adsorption to *E. coli* K-12 cells filamented by exposure to kanamycin, mitomycin C, and cefotaxime in comparison to untreated controls [55]. Furthermore, the relationship between phage adsorption and cell filamentation might differ based on phage receptor identity [54]. Davis et al. found that flagella-driven swimming motility and T4P-mediated twitching motility of *P. aeruginosa* PAO1 decreased upon aztreonam-lysine exposure in a concentration-dependent manner, suggesting that antibiotic treatment might result in decreased access to these motility structures as phage receptors [54]. Despite this hypothesis, synergy was still observed in planktonic killing assays conducted with this T4P-binding phage of PAO1 [54]. Therefore, the potential impact of changes to receptor availability on PAS was not elucidated. Nevertheless, these findings raise questions that suggest additional investigation of the impact of cell filamentation on phage receptor availability across different groups of phages should be considered. Together, these studies demonstrate that antibiotic-driven morphological changes may support phage adsorption, but the diversity of bacteriophage receptors adds nuance to this mechanism of PAS.

#### 2.1.3. Increased Phage Production as a Function of Lysis Rate and Burst Size

In addition to potential impacts on cell adsorption, increased virion production represents a hallmark of traditional PAS mechanisms that may be inherently linked to cell filamentation [46,53,54,55]. For instance, Kim et al. saw an increase in *E. coli* phage T4 burst size and a lengthened phage latent period in the presence of subinhibitory ciprofloxacin or cefotaxime [55]. Researchers found that T4 holins, which normally aggregate in the bacterial cytoplasmic membrane to form a channel that provides periplasmic access to phage lysis enzymes, aggregated more slowly in the expansive membrane of filamented cells [55]. The consequent delay in phage lysis enabled more assembled phage particles to accumulate before cell bursting occurred.

This mechanism of delayed lysis contrasts with the original description of PAS by Comeau et al., who found that cell lysis occurred faster in the presence of antibiotics [46]. Comeau’s proposed mechanism of accelerated lysis was further supported by the observation that holin-deficient phage mutants with delayed lysis rates demonstrated normal lysis in the presence of antibiotics [46]. Similarly, Davis et al. observed a shortened latent period for phages E79 and phiKZ in the presence of Aztreonam lysine, and it was hypothesized that faster lysis may increase overall phage production by quickening the rate at which cell lysis and the infection of neighboring cells by the released phage progeny occur [54].

Other works have noted increased burst size in morphologically altered cells without changes to phage replication kinetics [53,56], highlighting that antibiotic-driven increases in phage production occur through diverse pathways that may or may not be linked to cell size. In one study, the transcription of phage structural and lysis genes increased in the presence of antibiotics, suggesting that the upregulation of phage proteins may account for burst size increases in the absence of temporal changes to the phage infection cycle [56]. In another study, researchers tracked the localization of GFP-tagged phage DNA in elongated cells and found that the number of fluorescently labeled foci increased proportionally with cell length [53]. Based on this finding, researchers proposed that increased burst size is related to increased numbers of viral factories in elongated cells [53]. Overall, these studies show that increased phage production on a single-cell level through increased burst size or on a global level through accelerated lysis represent foundational mechanisms of PAS. However, the specific morphological or regulatory changes underlying this PAS mechanism of increased phage production are diverse and require further investigation.

### 2.2. Temperate Phage–Antibiotic Synergy and Resistance Trade-Offs

#### 2.2.1. Temperate Phage–Antibiotic Synergy (tPAS)

Following the injection of their genetic material into a bacterial host, temperate phages may engage in lytic growth or, through the action of early-expressed phage proteins, may integrate into the bacterial host chromosome, entering a reversible dormant state known as lysogeny [19]. The lysis–lysogeny switch is governed by a range of regulatory processes that are influenced by bacterial growth, environmental conditions, or other signals [76,77]. While the induction of the SOS response under antibiotic exposure can cause cell filamentation (see Section 2.1), for prophages present in the bacterial genome, SOS induction signals a threat to cell viability that stimulates excision and initiation of lytic growth, ultimately enabling phage-driven cell lysis [77]. In recent years, researchers have shown that the unique susceptibility of lysogens to SOS-inducing chemicals presents an opportunity for temperate phage-antibiotic synergy (tPAS) [57,58].

The term, tPAS, was first coined by Al-Anany et al. [58], who observed synergistic activity between the temperate *E. coli* bacteriophage HK97 and the SOS-inducing fluoroquinolone, ciprofloxacin. Investigation of the mechanistic basis of this synergy showed that 98% of the bacterial survivors of HK97 challenge tested positive for HK97 phage integration into the host chromosome, whereas a mere 32% of colonies isolated from combination treatments of HK97 with ½ MIC ciprofloxacin were lysogens. Additional experiments showed that the SOS-dependent induction of established HK97 lysogens by ciprofloxacin caused the observed synergistic effects. This mechanism of PAS has since been demonstrated for a range of other antibiotics including nalidixic acid, oxolinic acid, levofloxacin, mitomycin C, and trimethoprim [57].

Recently, researchers from the same group have uncovered a second, distinct mechanism of tPAS, in which protein synthesis inhibitors promote lytic replication of temperate phages independently of SOS activation [57]. Similarly to their original findings, the researchers found that treatment of *E. coli* with HK97 and sub-inhibitory gentamicin reduced the number of lysogens drastically from 92% in phage-only control groups to 2%. However, this reduction was not caused by phage induction as gentamicin treatment of HK97 lysogens at minimum inhibitory (MIC) or ½ MIC concentrations did not stimulate phage production. Evaluation of the frequency of lysogeny over time showed that antibiotic treatment reduced lysogeny at earlier timepoints than in their previous work [58]. This temporal change suggested that gentamicin biases temperate phages toward lytic growth during the critical lytic–lysogenic decision period that occurs between genome injection and the formation of established lysogens [57]. This mechanism has been demonstrated for several protein synthesis inhibitors, such as gentamicin, tetracycline, and azithromycin, and it is hypothesized that this group of antibiotics reduces the expression of phage proteins required to repress lytic growth during the early stages of phage infection [57].

Despite the conserved mechanism of ciprofloxacin-modulated effects on phage lysogeny across studies for *E. coli* [58] and *P. aeruginosa* [78], evidence suggests that the extent and mode of tPAS is variable between different compounds within antibiotic classes and for different temperate phage–antibiotic pairs [78]. Notably, Fatima et al. found that synergy between phage Cinder and piperacillin, a β-lactam, was accompanied by a decrease in the frequency of lysogeny, indicating that antibiotic treatment had influenced the lysis–lysogeny switch [78]. Conversely, synergy between the *Pseudomonas* phage Meadow and the β-lactam, meropenem, was not associated with the modulation of the lytic–lysogenic cycle, and instead, likely occurred through traditional PAS mechanisms [78]. Still, the discovery of tPAS represents a new mode of synergy that extends PAS beyond the traditional mechanisms of antibiotic-driven changes to phage adsorption, burst size, or replication dynamics, and highlights a potential new use for temperate phages in therapy.

#### 2.2.2. Phage-Driven Antibiotic Sensitization

Receptor mutation is widely considered a common mechanism of phage resistance [37], and has been shown to occur in vitro for phages recognizing a range of different receptors [36,44,79]. Although receptor loss prevents phage infection, it also provides a unique opportunity to significantly influence or steer the evolutionary trajectory of a pathogen toward a therapeutically beneficial phenotype [37]. In the context of phage–antibiotic synergy, the strategic use of phages recognizing structures conferring antimicrobial resistance may promote evolutionary trade-offs that potentiate the activity of otherwise ineffective drugs [44,80]. Indeed, this strategy has gained significant interest and examples of this process have been reviewed elsewhere [81,82,83]. Though precise mechanisms are rarely defined, the examples reviewed here point to phage-driven alterations to efflux or membrane permeability as the driving agents of re-sensitization.

The potential of efflux system-binding phages was first proposed upon the discovery of phage OMK01, which associates with outer membrane porin protein, OprM, during the infection of *P. aeruginosa* [59]. OprM comprises the outer membrane channel of two multi-drug efflux systems MexAB-OprM and MexXY-OprM, and researchers found that spontaneous phage-resistant *P. aeruginosa* mutants arising under OMK01 pressure were generally more sensitive to ciprofloxacin, tetracycline, ceftazidime, and erythromycin, likely due to reduced drug efflux capability [59]. These remarkable results were later supported by in vivo studies that found bacterial isolates recovered from phage- or phage and erythromycin-treated wax worms were more sensitive to erythromycin in comparison to untreated or antibiotic-only controls [43]. Examples of phage-driven antimicrobial sensitization have been reported for other MexXY-OprM-binding phages of *P. aeruginosa* [84] and for TolC-binding phages of *E. coli* [85], suggesting that phages recognizing efflux-system components could synergize with antibiotics extruded by those specific systems.

Additionally, phage-driven antibiotic sensitization to membrane-targeting antibiotics has been observed for phages recognizing cell envelope structures such as LPS [44] and capsular polysaccharide [80,86] positing increased membrane permeability or drug target accessibility as a potential evolutionary mechanism of PAS. This mechanism was recently hypothesized in *B. cenocepacia*, which produces a modified LPS that confers high levels of innate antibiotic resistance to certain membrane-disrupting drugs such as the LPS-binding polymyxin, colistin [44,87,88]. Ruest et al. found that phage-resistant *B. cenocepacia* K56-2 isolates obtained from infections with LPS-binding phages KS9, KS5, or JG068 showed various degrees of LPS-truncation [44]. These strains were more susceptible to colistin and to normalized human serum in comparison to wildtype controls, drawing a link between phage-driven alterations to the LPS and colistin sensitivity. In another study, resistance to *Acinetobacter baumannii* phage Phab24, which is thought to use capsular polysaccharide as its primary receptor, was found to arise through mutations in *gtr9*, a capsule-biosynthesis associated glycosyltransferase [86]. Similarly to the LPS mutants studied by Ruest et al., *gtr9* capsule mutants were more sensitive to colistin than their wildtype (WT) counterparts, and researchers proposed that the loss of *A. baumannii*’s capsular polysaccharide improves access for colistin to the outer membrane by reducing the physical barrier that may otherwise impede colistin activity [86]. Gordillo Altimerano et al. extended this model of sensitization in *A. baumannii* to include the cell wall targeting antibiotics ceftazidime and imipenem [60]. Specifically, phage-resistant mutants isolated against *A. baumanii* phage øFG02 in a mouse model of bacteraemia were resensitized to each of these antibiotics, to human serum, and to phage øLK01. These phage-resistant isolates were also found to have mutations in genes associated with capsule production, capsule export, and sugar production [60], implicating receptor loss as a driving factor or sensitization. More recently, phage-driven sensitization has been found to occur during poly-phage treatments of pan-resistant *P. aeruginosa* in mice [89]. Ashworth et al. found that a four-phage cocktail including LPS-binding phages PELP20 and 14/1 re-sensitized *P. aeruginosa* collected in mouse lung and liver tissues to meropenem [89]. Clear evidence of increased outer membrane permeability was observed in all re-sensitized *P. aeruginosa* variants isolated from the lungs of cocktail-treated mice, supporting improved drug diffusion as the mechanism of sensitization. Although genomic analysis of these phage-resistant variants showed limited evidence of LPS receptor mutation, researchers suggested that post-transcriptional changes in response to phage infection were responsible for the sensitization [89].

While each of these examples proposes a direct relationship between phage receptor loss and antibiotic susceptibility, instances of mutational heterogeneity in response to phage infection suggest that sensitization might also occur indirectly. For example, phage-resistant mutants of *P. aeruginosa* TL3780 arising from infection by phage vB3530, a predicted LPS-binding phage, showed phenotypic heterogeneity in regard to antibiotic sensitization [90]. Only 1 of 10 phage-resistant mutants were more susceptible to chlorhexidine and to the protein synthesis inhibitors gentamicin, amikacin, and tobramycin in comparison to WT. In contrast to whole-genome sequencing results for select antibiotic-susceptible mutants, which showed mutations in *wzy* encoding an LPS O-antigen polymerase, the antibiotic-sensitive strain contained several large-scale deletions, including in the LPS biosynthesis-related gene *galU*, the pigmentation synthase gene *hmgA*, and genes *mexX* and *mexY*, which are involved in the production of an aminoglycoside-inducible efflux pump. Accordingly, researchers hypothesized that the mutations to *galU* likely conferred phage resistance, whereas increased drug susceptibility could relate to reduced antibiotic efflux. Together these examples indicate that the application of phages targeting efflux-promoting or influx-limiting structures represents a promising new mode of PAS, but warn that phage resistance may arise through a range of diverse genetic changes that can make the precise mechanisms of re-sensitization unclear.

#### 2.2.3. Evolutionary Trade-Offs in Phage–Antibiotic Synergy

Although phage-driven re-sensitization represents the most well-established evolutionary mechanism of PAS, new research has identified shifts in the evolutionary trajectory of pathogens in response to combination treatments that uniquely support PAS. Specifically, Qin et al. found that the incubation of *Klebsiella pneumoniae* with phage H5 alone produced phage-resistant mutants with increased metabolic activity and competitiveness in comparison to untreated controls [56]. These mutants, which arose through missense mutations in *wcaJ*, a gene involved in capsule production, were more sensitive to ceftazidime than WT controls, consistent with the re-sensitization mechanism of PAS described earlier (Section 2.2.2). In contrast, dual H5 and antibiotic treatment also produced phage-receptor deficient mutants, but through mutations in *galU*, a UTP-glucose-1-phosphate uridylyltransferase involved in the biosynthesis of several cell structures such as LPS and capsule. Unlike the mutants selected under phage-only conditions, these isolates were more resistant to ceftazidime than WT controls but demonstrated collateral defects in growth and competitiveness. Researchers proposed that the fitness trade-offs obtained as the collateral cost of simultaneous phage and antibiotic resistance support treatment efficacy and represent a new means of PAS.

Similarly to Qin et al. [56], Parab et al. [61] found that the addition of antibiotics changes the course of bacterial evolution; however, instead of imposing unique fitness costs on bacterial survivors of combination treatment, they found that antibiotics suppress phage resistance by biasing against the proliferation of certain types of phage-resistant LPS phenotypes [61]. In flux experiments, researchers found that while resistance to phage ϕX174 arose via mutation to either rough or deep rough LPS genes, most frequently, it was the result of deep rough gene mutations. Researchers speculated that this overabundance of deep rough mutants may reflect a disproportionate number of mutational targets that produce deep rough LPS in comparison to rough LPS, or could be due to differences in the extent of phage resistance conferred by various LPS mutant types. However, upon the addition of chloramphenicol at a concentration of 2 ug/mL, the phage-resistant population was biased toward rough LPS mutations 100% of the time. A similar, albeit smaller effect was observed for gentamicin. Together with the observation that total phage resistance arose much less frequently in phage antibiotic combination treatments, researchers proposed that antibiotic-induced bias against rough LPS mutations reduced the number of possible phage-resistance mutation types that can proliferate under combination treatment pressure, ultimately suppressing phage resistance [61]. These studies suggest that PAS does not rely solely on direct modulation of phage infection dynamics or phage-driven sensitization to antimicrobials; it can also steer bacterial evolution toward less fit states or block resistance pathways under combined phage-antibiotic pressure. Overall, it seems that PAS is governed by direct antibiotic or phage-promoted mechanisms, but also by the cumulative effect of coordinated evolutionary pressures.

### 2.3. Other Potential Synergistic Effects

#### 2.3.1. Deep Dormant Cell Resuscitation

Under stress conditions, some bacterial cells enter a state of dormancy, where cell functions required for replication and division temporarily cease [91]. Given that phages often rely on host cell replication machinery and antibiotics target life processes, this state of dormancy renders these cells, sometimes termed “persisters”, antibiotic-tolerant and prevents infection by many phages [62,92]. A recent study found that *P. aeruginosa* jumbo phage, Paride, infects deep dormant, antibiotic-tolerant cells in a process that requires stationary-specific host stress signals (p)ppGpp and RpoS [62]. Synergistic activity against the deep dormant cultures of *P. aeruginosa* PAO1 was seen for combination treatment with the phage Paride and meropenem, both in vitro and in a tissue cage mouse model of chronic *P. aeruginosa* implant infection. While the exact mechanism of synergy requires further investigation, experiments showed that Paride re-sensitizes deep dormant cultures to meropenem, and researchers proposed that intracellular contents such as signaling molecules or nutrients, or cell wall components released from Paride-lysed cells may resuscitate other dormant cells in the culture, reducing antibiotic tolerance. In theory, this mechanism of action may extend to other antibiotic classes beyond the cell wall targeting β-lactamases. However, no synergistic activity was seen for Paride in combination with ciprofloxacin or tobramycin, leading researchers to speculate that certain classes of antibiotics may inhibit phage DNA replication, reducing efficacy. Notably, Paride is genetically unrelated to the nucleus-forming jumbo phage phiKZ [62], and the ability to lyse dormant cultures does not appear to be generalized across all jumbo phages. Also, non-jumbo phages active against dormant *P. aeruginosa* PAO1 cells have been characterized [93], but their potential for PAS has not been investigated. Nonetheless, these findings shed light on a new potential mechanism of PAS and highlight the need for additional investigation of PAS strategies targeting persistent infections.

#### 2.3.2. Purified Phage Proteins as Antimicrobial Adjuvants

As an alternative to phages, phage-derived lysins, which are peptidoglycan (PG) hydrolases that degrade bacterial cell walls by cleaving specific bonds in the peptidoglycan matrix, have gained significant traction and are being investigated for treating priority pathogenic bacteria [5,94]. During the early stages of phage infection, virion-associated lysins aid in clearing peptidoglycan to assist phage genome injection [94]. Endolysins, on the other hand, are produced within the host cell during later stages of phage replication, and play a crucial role in phage-mediated host lysis [94]. Resistance to phage lysins is rare, making them an attractive alternative to whole phage particles in therapy [94].

In Gram-positive bacteria, which harbor a thick peptidoglycan layer that protects the cell membrane, phage lysins may potentiate access of certain antibiotics to their cellular targets. For example, exebacase (formerly known as CF-301), is a phage-derived lysin that has been extensively researched as an antibiotic adjuvant for Gram-positive *S. aureus* infections [63,95,96] and has been the subject of several recent clinical trials [97,98]. Exebacase has been shown to increase antibiotic binding of both daptomycin and vancomycin to *S. aureus* cell envelope structures, and is hypothesized to support antibiotic activity by degrading peptidoglycan, improving cell permeability and access to antibiotic targets [63]. Synergistic effects for exebacase have also been seen in combination with cell wall targeting antibiotics in checkerboard assays conducted across a range of methicillin-resistant and methicillin-sensitive *S. aureus* strains with oxacillin, cefazolin, telavancin [95]. Furthermore, synergistic or additive effects have been seen for combinations of exebacase with clindamycin, azithromycin, linezolid, gentamicin, or sulfamethoxazole-trimethoprim, suggesting that exebacase-driven antibiotic potentiation is not limited to cell wall- or membrane-targeting drugs [95]. Indeed, combination treatment with antibiotics and phage-derived lysin P128 has similarly been found to re-sensitize drug-resistant strains of *Staphylococcus epidermidis* and *S. aureus* to vancomycin, oxacillin, cephazolin, daptomycin, linezolid, and ciprofloxacin [99], which target several different cellular processes. Despite the significant success of antibiotic–lysin combination therapies in vivo, including in a murine model of *Staphylococcal* bacteremia [63,99], and in a neutropenic murine thigh model [96], the failure of a recent phase 3 clinical trial using exebacase alongside clinically relevant antibiotics including daptomycin [98] suggests that additional work is required to perfect this strategy against Gram-positive pathogens.

Unlike in Gram-positive bacteria where the peptidoglycan layer is exposed to the extracellular environment, the peptidoglycan of Gram-negative bacteria is encased by an outer membrane, which prevents the activity of many purified phage lysins (reviewed in ref. [94]). Thus, it is reasonable to predict that combination treatments of lysins and membrane-permeabilizing antibiotics may offer an effective strategy to combat Gram negatives. Indeed, several studies have identified synergy between lysins and colistin, a membrane permeabilizing polymyxin antimicrobial [100,101]. However, the mechanism of synergy may be more complex than simple lysin-mediated outer membrane penetration of the antibiotic. For instance, although the lysin LysMK34, derived from *A. baumanii* phage MK34, synergizes with colistin, a lack of synergy with closely related membrane-disrupting drug, polymyxin B, suggested that beyond the cyclic heptapeptide structure shared by each drug, the fatty acid tail present on colistin but not polymyxin B, promotes synergy through a separate mechanism [101].

To overcome the Gram-negative outer membrane, recombinant endolysins with improved cell wall permeabilizing activity have been developed, though knowledge about how these modified lysins interact with antibiotics remains limited. Investigation of LNT113, an engineered endolysin produced by fusing the antimicrobial peptide cecropin A to a mutant version of the endolysin produced by *E. coli* phage PBEC131, showed synergy with colistin in checkerboard assays [102]. In contrast, modification of LysMK34 from *A. baumanii* phage MK34 with cecropin A downgraded the interaction with colistin from synergistic to additive, possibly due to redundancy of outer membrane permeabilization function by colistin and the fusion protein [103]. Aside from lysins, phage-derived depolymerases that degrade carbohydrate components of the cell envelope such as LPS, capsule, or extracellular polysaccharides (EPS) [94], have been shown to synergize with colistin by degrading the bacterial capsule of *A. baumannii*, allowing colistin to better access the outer membrane [64]. Other recent examples of strain-dependent synergy via antibiotic sensitization between phage-derived depolymerases and gentamicin, levofloxacin, and meropenem have been described in *K. pneumoniae*, although the precise mechanism of action remains unclear [104].

These studies highlight that phage-derived proteins may synergize with antibiotics by improving access to antibiotic targets, through direct antimicrobial activity, or through other uncharacterized mechanisms. Although the potential applications for phage-derived proteins toward reducing AMR infections are extensive and have gained significant interest for Gram-positive infections, this nascent strategy against Gram-negative bacteria requires additional development

## 3. Phage–Antibiotic Antagonism

**Figure 2:**
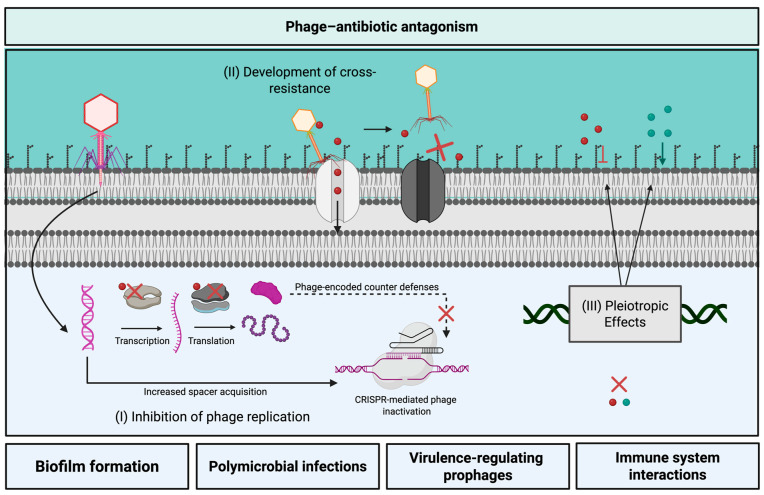
Potential negative impacts on phage–antibiotic treatment efficacy. Known mechanisms of phage–antibiotic antagonism include (**I**) inhibition of phage replication by antibiotics targeting translation and transcriptional machinery [108], increased CRISPR-mediated immunity through improved spacer acquisition in the presence of bacteriostatic antibiotics [109], and reduced production of anti-CRISPR counter-defenses [110]. (**II**) Cross-resistance may occur as a consequence of phage–antibiotic combinations with shared bacterial structures for phage binding and antibiotic uptake [111]. (**III**) Mutations that confer resistance to phages and result in antibiotic sensitization to certain drugs may pleiotropically increase resistance to other antibiotics [85]. As described in Section 4.3, other potential effects that could increase antibiotic resistance, alter pathogen virulence, or dictate treatment efficacy are increased biofilm formation, off-target induction of virulence-regulating prophages, and interactions with human immunity factors. Colored spheres represent antibiotics. Created with BioRender.com.

While the advances toward understanding phage–antibiotic synergy described above could unlock new strategies for targeting AMR pathogens, antagonism between phages and antibiotics has also been reported [105,106,107]. Despite the potential impacts of phage–antibiotic antagonism on treatment outcomes, little is known about the mechanistic basis of these interactions, and antagonism has been identified as a major knowledge gap in the field [50]. In this section, we review the current state of phage–antibiotic antagonism (Figure 2) and highlight areas that require additional investigation.

### 3.1. Possible Mechanisms of Phage–Antibiotic Antagonism

Given that many phages rely on bacterial machinery for central dogma processes, phage–antibiotic antagonism may occur as the direct result of reduced phage replication by antibiotics targeting cellular machinery, such as bacterial RNA polymerase or bacterial ribosomes [105,108,112]. This phenomenon has been observed for several specific phage–host pairs, and examples have previously been reviewed elsewhere [108]. Though many early examples of phage antagonism were due to RNA polymerase inhibitors such as rifampicin [108], DNA gyrase or topoisomerase inhibitors such as nalidixic acid have also been found to inhibit the replication of some phages [113]. More recently, reports of antagonism between phages and protein synthesis inhibiting antibiotics have surged, and have been shown to span a range of diverse phage–host pairs [105,106,107]. For example, Kever et al. found that protein synthesis inhibiting aminoglycosides antagonized the activity of several phages infecting *E. coli, Streptomyces venezuelae*, and *Corynebacterium glutamicum*, and further investigation found that this inhibition occurred during the early stages of phage replication [105]. Another recent study reported antagonism between various protein synthesis inhibitors and phages infecting *S. aureus, Enterococcus faecium*, or *P. aeruginosa*, citing a decrease in burst size for some, but not all, antagonistic pairs [106]. These findings propose that protein synthesis inhibitors may be especially prone to antagonizing phage activity.

Separate from the direct inhibition phage replication, new data suggests that certain groups of antibiotics may antagonize phage activity by bolstering antiphage defenses and reducing the expression of phage-encoded counter-defenses [109,110]. CRISPR-mediated resistance to phage infection begins with the acquisition of spacers derived from invading phage nucleic acids. These spacers are integrated into the bacterial chromosome at the CRISPR array, which is a heritable anthology of infectious DNA sequences that confers adaptive immunity to invading nucleic acids [114]. Integrated spacers are subsequently transcribed and processed into mature crRNA’s that guide nucleolytic Cas proteins to cleave nucleic acids matching the spacer sequences [114]. Crucially, in order to protect against phage infection, spacer acquisition, integration, and expression must occur quickly enough to outpace phage DNA replication and cell lysis. Recently, researchers found that several bacteriostatic antibiotics increased CRISPR immunity to phage DMS3vir in *P. aeruginosa* PA14 [109]. In particular, trimethoprim, chloramphenicol, tetracycline, and erythromycin led to increases in CRISPR immunity against DMS3vir. Results showed that with the exception of erythromycin, all antibiotics negatively impacted phage production, delaying the eclipse period and slowing phage production, which provided time for increased spacer acquisition [109]. This phenomenon was restricted to bacteriostatic antibiotics, as bactericidal antibiotics streptomycin, gentamicin, carbenicillin, and ciprofloxacin did not lead to large increases in CRISPR immunity [109]. Other work has shown that antibiotic interference with the expression of phage-encoded anti-CRISPR proteins (Acr), may reduce phage infection by preventing phage counter-defenses from effectively inactivating CRISPR machinery. Specifically, treatment with chloramphenicol, erythromycin, and tetracycline, enabled CRISPR-mediated immunity of PA14 against DMS3vir carrying the *acr* gene, *acrIF1* [110]. Notably, researchers found that gentamicin, which also works by targeting protein synthesis, reduced phage activity to similar levels in the absence or presence of *acrIF1* in both CRISPR knockout and CRISPR immune PA14 cells [110]. This CRISPR and Acr-independent antagonism observed for gentamicin highlights the idea that antibiotics targeting the same cellular processes may have diverse antagonistic activities.

In addition to these antibiotic-mediated effects on phage infection, the evolutionary consequences of phage predation may increase antibiotic resistance in certain systems. For *E. coli* phages T6 and U115, which bind Tsx, a key porin required for specific antibiotic uptake, phage receptor loss may increase bacterial resistance to the peptide antibiotic, albicidin [111]. Spontaneous resistant mutants arising from T6, U115, or albicidin treatment harbored mutations in *tsx* resulting in resistance to all three antimicrobials [111]. Unlike other studies that identified fitness trade-offs in phage and antibiotic-resistant variants [56], these mutants showed no fitness defects in vitro [111]. While selection under combined treatment pressure was not investigated, these findings imply that bacterial structures enabling both phage binding and antibiotic uptake may hinder the effective penetration of either antimicrobial. Similarly, phage–antibiotic antagonism may occur through pleiotropic effects of phage-driven mutation [85]. Burmiester et al. found that *E. coli* phage U13B, which recognizes both TolC the outer membrane component of the TolC-AcrAB efflux pump and LPS as receptors, selected for mutations in both structures. Although TolC mutants generally demonstrated increased sensitivity to the TolC-AcrAB substrate tetracycline, and LPS mutants were more sensitive to the antimicrobial polypeptide colistin, several LPS mutants also demonstrated increased resistance to tetracycline [85].

Beyond these established examples of phage–antibiotic antagonism, it is possible that phage-driven changes to bacterial biofilms could negatively affect treatment efficacy in some situations and require consideration. Specifically, although the evidence is limited, some reports indicate that phage predation at low concentrations may inadvertently increase biofilm formation. This phenomenon was reported by Hosseinidoust et al. who found that treatment with certain bacteriophages could increase biofilm formation for *P. aeruginosa*, *Salmonella enterica* Typhimurium, and *S. aureus* [115]. More recently, sub-lethal amounts of lytic phage phiIPLA-RODI have been shown to increase *S. aureus* biofilm formation [116]. Transcriptional analysis of phage-treated biofilms found an increase in genes related to the bacterial stringent response. While researchers did not examine the antibiotic susceptibility of phage-treated biofilms in comparison to normal biofilms, they suggest that both the changes to biofilm formation and uptick in stringent response gene expression under phage predation could potentially enhance antibiotic resistance [116]. To date, increased biofilm formation under low levels of phage predation has not been directly linked to increased antibiotic resistance, but, considering the antibiotic-tolerant nature of bacterial biofilms, this area of research warrants investigation.

Together, these studies demonstrate that phage–antibiotic antagonism can arise through diverse and sometimes unexpected mechanisms including reduced phage replication, disrupted counter defenses, and altered bacterial surface structures. These effects highlight the need for additional mechanistic investigations of phage–antibiotic antagonism and careful evaluation of phage–antibiotic combinations to avoid unintended treatment failures.

### 3.2. Challenges Toward Predicting Phage–Antibiotic Antagonism

#### 3.2.1. Exceptions Are the Rule: Antibiotics

Although it is tempting to draw broad conclusions about the usefulness of certain drug classes for PAS based on their mechanisms of action, exceptions prevent the polar categorization of antibiotics as synergistic or antagonistic. For example, despite reports of antagonism with *P. aeruginosa* phage DMS3vir [110], PAS has been demonstrated using the ribosome-targeting antibiotic, gentamicin against both monocultures of *P. aeruginosa* and in mixed-species biofilms of *P. aeruginosa* and *S. aureus* [106,117], highlighting that protein synthesis inhibitors should not be discounted for PAS. Additionally, antagonism between polyvalent phage SaP7 and β-lactams has been reported in both mouse and piglet models of *Salmonella* infection [118], proving that antibiotics that do not directly inhibit central dogma processes may also interact antagonistically with certain phages. Opposing reports about the antagonistic or synergistic activities of other antibiotics such as the cell wall disruptor colistin, have been observed across phage–host systems [86,119].

The rules are further muddied by nuanced structural or mechanistic differences between drugs of the same class. Kever et al. found that *Streptomyces venezuelae* phage Alderaan was inhibited by hygromycin and apramycin, but not by the related aminoglycoside, spectinomycin, despite antiviral activity observed for spectinomycin against other tested phages [105]. Similarly, Jiang et al. showed that mycobacteriophages D29 and phAE159 were inactivated by kanamycin, hygromycin, and streptomycin, but spectinoymicin did not produce antiviral effects [112]. Jiang et al. hypothesized that an amino acid sugar moiety, which was absent in spectinomycin but present in the antiviral aminoglycosides, may play a role in inhibiting viral DNA replication [112]. In another study, *A. baumanii* phage Indi synergized with ceftazidime, but was antagonized by piperacillin–tazobactam [120], suggesting that differences in phage–antibiotic interactions exist across β-lactams. These examples highlight that any delineation between antagonistic and synergistic antibiotic groups made based on current reports is a preliminary guideline and exceptions are the rule.

#### 3.2.2. Antagonism Depends on the Specific Phage–Host System

Adding to the nuanced impact of antibiotic class on the phage–antibiotic relationship, antagonism may also depend on specific phage characteristics. For example, *Ralstonia solanacearum* jumbo phages ΦRP12 and ΦRP31 are capable of replicating in the presence of 20 ug/mL rifampicin [23], whereas closely related jumbo phages ΦRSL2 and ΦRSF1 are inhibited by 3–5 ug/mL rifampicin [23,121]. Despite these findings, genetic characterization and analysis of ΦRSF1 virion-associated proteins identified a full complement of phage-encoded RNA polymerase subunits [121], which postulates that phage-encoded transcriptional machinery may also be affected by certain antibiotics. In other work conducted in ExPEC strain JJ2527, phages ΦHP3 and ΦES12 showed unique patterns of antagonism across phage and ciprofloxacin concentrations despite sharing 98% genomic similarity [122]. Researchers noted a 6-fold difference in burst size between these phages, suggesting that genetically similar phages may exhibit distinct infection kinetics [122]. Adding to the variable effects seen for similar phages, other works suggest that antagonism varies greatly across phage–host pairs. A recent study found that rifampicin combined with diverse phages recognizing a range of different bacterial receptors synergistically killed *P. aeruginosa* strain PA14, but similar experiments conducted in *S. aureus* strain 1203 resulted in antagonism for all phage–rifampicin combinations [40]. Furthermore, during a survey of aminoglycoside inhibition across phages infecting *E. coli*, *Streptomyces coelicolor*, *Streptomyces venezuelae*, and *Corynebacterium glutamicum*, Kever et al. identified inhibitory activity for at least one aminoglycoside against a diverse range of dsDNA siphophages, but did not detect inhibition amongst the tested RNA or filamentous phages in their study [105]. Cumulatively, these studies suggest that bacteriophages that encode their own transcriptional machinery may be more likely to avoid antagonism with transcription-inhibiting antibiotics [23], but small differences between related phages or broader differences between groups of bacteria may significantly impact phage–antibiotic interactions and the patterns driving antagonism across different groups of phages remain unclear.

#### 3.2.3. In Vitro Differences Across Concentration, Time, and Condition

For a single phage–antibiotic pair, antagonism may further depend on specific stoichiometric [122] or temporal [123] parameters, and may be influenced by the testing conditions [122]. Gu Liu et al. showed that antagonism between 4 ug/mL colistin and phage ΦHP3 occurred at phage densities of 10^8^ PFU/mL, but not at 10^9^ PFU/mL [122]. A similar effect was seen for chloramphenicol (4 ug/mL) when phage was added at 10^5^ PFU/mL, but not at other tested phage densities [122]. In long-term evolution experiments, Torres-Barceló et al. found that treatment with the macrolide erythromycin sometimes exerted a negative impact on phage virulence after 2 days of antibiotic exposure, but not at later timepoints [123]. Other work found that combination treatment with phage ΦJB10 and increasing concentrations amikacin, ciprofloxacin, and meropenem was less effective against PAO1 in comparison to phage-only and antibiotic-only controls at 4 h, but not at 22 h [124]. In this study, further testing of each phage–antibiotic combination in biofilms did not detect any signs of phage–antibiotic antagonism [124]. Furthermore, Gu Liu et al. found that combinations of ceftazidime and ΦHP3 in human urine or heat-inactivated human serum showed antagonistic activity, which was likely due to growth under these ex vivo conditions [122]. These examples suggest that phage–antibiotic interactions are dynamic and sensitive to a range of complex factors. This variability across conditions urges that rigorous testing of phage–antibiotic pairs across a range of treatment-relevant conditions is needed to develop effective phage–antibiotic strategies, as described next.

## 4. Development of Phage–Antibiotic Strategies

### 4.1. In Vitro Evaluation of Phage–Antibiotic Pairs

**Figure 3:**
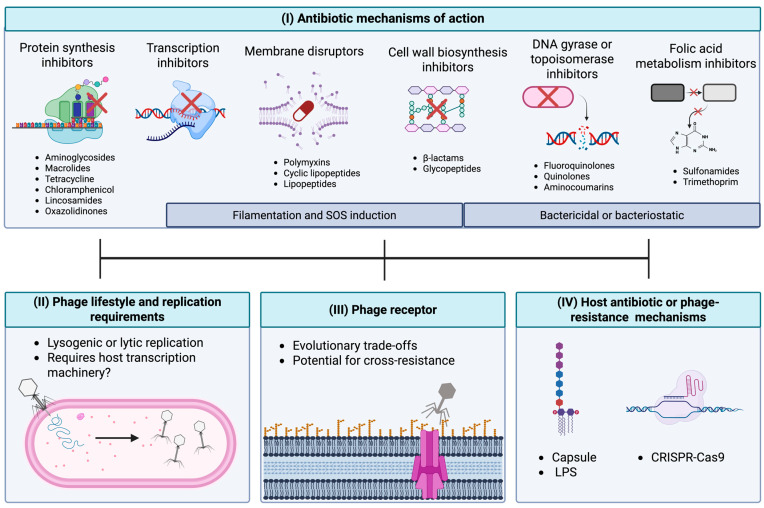
Considerations toward formulating effective phage–antibiotic pairs. (**I**) The general mechanisms across antibiotic classes are distinct [72], and may shed light on their potential synergistic or antagonistic effects with specific phages. For instance, antibiotics known to induce cell filamentation may be more likely to produce synergy with certain phages than antibiotics that do not [53]. On the other hand, examples of antagonism are common for transcription inhibitors [108] or protein synthesis inhibitors [105,106,107]. However, the unique biology of characterized phages available for use in PAS treatments also contributes to their compatibility with certain antibiotics (**II**). Phages that encode their own RNA polymerases may be immune to antagonistic effects caused by transcription-inhibiting antibiotics such as rifampicin [23]. Also, lysogeny-capable phages may be more likely to synergize with protein synthesis inhibitors or SOS-inducing antibiotics due to the unique interaction of these antibiotics with lysogeny-associated processes [57,58,78]. (**III**) The receptor recognized by certain bacteriophages may help researchers to anticipate phage-driven evolutionary changes that could lead to antibiotic sensitization or synergistic effects. For instance, the use of LPS or capsule-binding phages with membrane-disrupting antibiotics, or efflux pump-binding phages with antibiotics that act as substrates for the phage-bound efflux system may improve treatment efficacy by promoting phage resistance trade-offs that improve target accessibility or decreasing drug extrusion [44,59,80]. Furthermore, phages recognizing the same receptor may be more likely than taxonomically similar phages to demonstrate similar patterns of synergy across antibiotic classes [40]. Thus, the receptors recognized by phages in known synergistic phage–antibiotic pairs could aid in the identification of additional treatment pairs for that host. (**IV**) In the same way that phage receptor identity might allow researchers to predict effective phage–antibiotic pairs, knowledge of intrinsic resistance features such as modified LPS or capsule production informs which types of evolutionary trade-offs might be possible in certain pathogens [44,59,80]. Also, bacteriostatic antibiotics have been shown to support CRISPR-mediated phage defense systems in *P. aeruginosa*, suggesting that bactericidal antibiotics may be more likely to produce PAS in some CRISPR-competent hosts [109]. Created with BioRender.com.

Together, the known mechanisms of phage–antibiotic synergy and antagonism insinuate that integrated consideration of antibiotic mechanisms of action, phage biology, and pathogen biology may aid in identifying effective phage–antibiotic combinations (Figure 3). Yet, the challenges toward predicting antagonism outlined earlier (Section 3.2) necessitate that diligent in vitro screening of phage–antibiotic combinations across an array of concentrations, timepoints, and media types is required for all potential phage–host pairs. However, the absence of a standard screening protocol for testing phage–antibiotic pairs is an issue that has been pointed out by several researchers [125,126]. Here, we highlight the most common in vitro techniques used to assess PAS.

#### 4.1.1. Agar-Based Methods

In one of the earliest examples of phage–antibiotic synergy, Comeau et al. combined a standard disk diffusion assay, used to detect antimicrobial susceptibility with a double agar overlay used to enumerate phage, producing an elegant method to detect PAS [46]. In that foundational work, antibiotic-impregnated disks were placed on the surface of a double agar overlay of bacteria and phage, and plaque size was measured as an indicator of enhanced phage replication in zones containing diffuse antibiotics [46]. Other variations in the double agar overlay that involve adding the antibiotic directly to the soft agar and/or base agar rather than on disks, and counting the total number of plaques produced in the presence of antibiotic as well as plaque diameter have been investigated [127]. Comparative testing of these different variations with a panel of 43 diverse antibiotics indicated the detection assay may require optimization based on antibiotic identity [127], further complicating the development of a standardized methodology.

Although double-layer agar methods served as the basis for the development of PAS as a field, they are largely qualitative tools and may not detect synergistic interactions that do not directly modulate phage replication, such as phage-driven drug sensitization. More recently, researchers have developed a quantitative agar-based methodology for detecting PAS based on zones of bacterial clearance rather than plaque formation [126]. In this approach, filter paper strips soaked with phage or antibiotic are placed onto a lawn of the target pathogen at a right angle, and computational modeling is used to determine the type of interaction between antimicrobials based on the lysis patterns found on areas of the lawn distal to each strip [126]. This technique was validated for multiple phage–antibiotic pairs against multiple strains of Gram-positive species including vancomycin-resistant isolates of *E. faecium* and *Enterococcus faecalis*, and across several strains of the Gram-negative pathogen *Stenotrophomonas maltophilia*. Notably, synergy was observed between phage *Bob* and ampicillin or vancomycin against intrinsically β-lactam-resistant, or vancomycin-resistant strains of *E. faecium*, respectively, indicating that this methodology may detect non-traditional PAS interactions such as drug sensitization [126].

One foreseeable limitation of agar-based methods is that they may not accurately measure PAS for phages that do not infect readily in solid matrices. For example, jumbo bacteriophages that encode large genomes of 200 kb or more, produce large virions that may not diffuse easily through solid media [128], which could potentially limit the detection of PAS on solid plates. Poor diffusion may also present a barrier to the detection of PAS for certain drugs. In a recent study, ciprofloxacin and oxacillin only produced small changes in the plaque size of *S. aureus* phage vB_Sau_S90, while vancomycin did not affect plaque size at all [129]. Yet, all three antibiotics were found to synergize with vB_Sau_S90 in subsequent liquid assays. In that work, researchers proposed that poor drug diffusion and the innate antibiotic-resistance status of bacterial isolates may affect PAS detection on agar plates [129]. Other studies have noted significant differences in phage infectivity in solid vs. liquid media types [33]. Thus, while the accessibility of a simple agar-based technique makes it an attractive option for clinical laboratory use [126], these methods may be prone to false negatives.

#### 4.1.2. Broth-Based Methods

As an alternative to agar-based methods, microtiter-plate checkerboard assays are a standard method for testing antimicrobial synergy [49] that are commonly adapted for testing phage–antibiotic pairs [58,106,122,125]. In general, these assays compare bacterial viability or growth across a checkerboard of decreasing concentrations of antibiotic tested in combination with decreasing MOI of phage. While the overall set-up of experiments is similar across studies, several different methods exist to quantify the antibacterial effects. For example, some studies have used colorimetric shifts produced through the bacteria-mediated reduction of 2,3,5-triphenyltetrazolium chloride (TTC) to red formazan as an indicator of bacterial viability in each treatment condition [125]. Alternately, spectrophotometric measurements of cell turbidity are commonly used to measure bacterial density [58,122].

Although checkerboard assays are widely popular, they are limited by their reliance on static measurements taken at specific timepoints [49,130]. Therefore, kill curves, which measure the dynamic growth of a pathogen over time are preferable in some situations [130]. As such, it has become increasingly common for researchers to use both methods in their work [58,122]. For example, synography, which was introduced by Gu Lui et al., provides a bridge between traditional checkerboard assays and dynamic kill curves [122]. Like checkerboards, these experiments are conducted in microtiter plates across a range of phage and antibiotic concentrations, and bacterial growth is measured as the optical density in each treatment well [122]. These measurements are taken at 15 min intervals across a 24 h period and are compared to absorbance values gathered from growth controls to generate a (%) reduction value for specific timepoints. In addition to allowing for treatment comparisons at a range of endpoints, the frequent measurements provide dynamic kill curve data that allows for additional quantitative metrics, such as the area under the curve (AUC), to be calculated and compared as needed. Synography has been adopted by several research groups [60,120,124], and has also been used to detect PAS in treatment-relevant conditions, such as in human blood and urine [122].

While broth-based methods offer a simple, relatively fast way to produce quantitative datasets, other researchers have pointed out that they require specialized reagents, skills, and spectrophotometers that may not be accessible to all clinical facilities or research groups [49,126]. Furthermore, spectrophotometric quantification methods may be affected by cell agglomeration, morphological heterogeneity in mixed cultures, or the presence of light-absorbing bacterial products [131], complicating the standardization of broth-based methods across systems. Adding to these challenges, the mathematical strategies used to quantify antimicrobial effects and determine synergy, additive effects, and antagonism vary across studies [106,122,125,132]. Indeed, widespread adoption of a standardized methodology for collecting, analyzing, and interpreting PAS data could help uncover patterns of phage–antibiotic synergy and antagonism across antibiotic classes and phage–host systems. Standardized testing protocols could also support investigations of the impact of treatment sequence on PAS, which represents a major consideration toward the development of combination treatments, as described in the next Section 4.2.

### 4.2. Timing of Application

As other reviews have noted [52], the efficacy of treatment orders where both antimicrobials are applied simultaneously, phages are applied prior to antibiotic, or phages are applied after antibiotic, may correspond with the mechanism of PAS or antagonism between phage–antibiotic pairs. Several studies suggest that phage pre-treatment followed by antibiotic treatment after a short delay may improve outcomes for phage–antibiotic combinations prone to antagonistic effects [133,134]. For instance, simultaneous treatment on *P. aeruginosa* PA14 biofilms with phages NP1 and NP3 in combination with gentamicin or tobramycin was no more effective than antibiotic alone, likely due to antibiotic-mediated inhibition of phage infection [133]. In contrast, sequential application of either antibiotic 24 h after phage treatment significantly decreased bacterial density relative to single antimicrobials [133]. In another study, simultaneous treatment of *S. aureus* biofilms with phage SATA-8505 produced antagonism with cell wall biosynthesis inhibitor, cefazolin and concentration-dependent antagonism with vancomycin, whereas sequential treatment with phage followed by vancomycin, cefazolin, linezolid, or tetracycline after 24 h produced synergistic or additive antimicrobial effects [134]. In the context of antagonism, early phage treatment could allow for meaningful levels of phage replication to progress prior to the onset of antibiotic-mediated antagonism [135]. These results could also reflect the biofilm-specific mechanism of PAS, where the initial phage-mediated biofilm destruction enables improved antibiotic penetration [129]. In support of this theory, Loganathan et al. found that out of all possible treatment orders, oxacillin followed by phage was most effective against planktonic *S. aureus* in checkerboard assays, but pre-treatment with phage best supported survival of *Galleria mellonella* waxworms infected with *S. aureus* biofilms [129]. Thus, both the antibiotic mechanism of action and the capacity of pathogens to form biofilms may impact the treatment sequence.

Whereas these examples argue a strong case for applying phages prior to antibiotics, other works suggest that the benefits of simultaneous application may outweigh the drawbacks [117]. Despite the potential antagonistic effects of gentamicin on phage replication, Akturk et al. found that multiple rounds of simultaneous treatment with phages EPA1 and SAFA and gentamicin more effectively cleared polymicrobial *P. aeruginosa* and *S. aureus* biofilms than phage-first sequential treatment strategies [117]. Researchers speculated that simultaneous pressure may limit the evolution of phage and antibiotic-resistant bacteria better than sequential treatments [117]. This potential evolutionary benefit has been supported in other studies which found that pre-treatment of *A. baumanii* with phage vB_AbaM-IME-AB2 8 h prior to colistin was more effective than simultaneous treatment in checkerboard assays [136]. However, despite colistin-mediated antagonism of phage replication, simultaneous treatment was shown to suppress the emergence of phage resistance more effectively than other treatment orders [136]. Though the underlying mechanism of suppression in these studies is unclear, it is possible that simultaneous application could suppress resistance by biasing against the evolution of certain types of phage resistance mutations, as discussed in Section 2.2.3 [61]. Similarly, in phage-cocktail studies, bacterial resistance to two sequentially applied phages arose in a stepwise manner via multiple mutations to phage receptor-associated genes, whereas simultaneous phage treatment led to single mutations or large-scale rearrangements that conferred lower levels of phage resistance and imposed significantly higher fitness costs to bacteria [137]. While the mechanisms of action of antibiotics impose distinct evolutionary pressures in comparison to phages, these studies highlight the potential evolutionary benefits of tandem antimicrobial application.

Although these studies indicate that both phage-led and simultaneous strategies may be effective under certain circumstances, the success of antibiotic-led treatment modes has been mixed. Some studies have seen excellent success for PAS strategies of antibiotic pre-treatment in checkerboard assays, but slightly reduced efficacy of this modality in vivo [129]. Others have noted that the application of gentamicin prior to phages was generally less effective than other treatment orders in biofilm assays [117]. Experiments conducted with temperate phage Hali showed that simultaneous treatment, and sequential treatment of phage followed by ciprofloxacin after 4 h resulted in a similar level of growth reduction in *P. aeruginosa* [78]. However, when an antibiotic was added 4 h prior to phage treatment, the killing effect was reduced, and researchers have suggested that the rate of lysogeny could bear importance in determining treatment order [78]. Moreover, other studies posit that treatment order may be impacted by bacterial growth dynamics [125]. Phage–antibiotic synergy between *Pseudomonas* phage JG024 and ciprofloxacin was observed upon simultaneous application of phage and antibiotics, and in cases where a short delay occurred after the administration of one antimicrobial, but not if there was a long (6 h) delay [125]. In this case, researchers suggest that if bacteria are grown under subinhibitory concentrations of phage or antibiotic for too long, their accumulated bacterial density may be too high to allow for meaningful clearance. Together, these findings suggest that phage-led, simultaneous, and antibiotic-led treatment orders may all be effective under certain circumstances, but indicate that several factors including antibiotic mechanism of action, or the rates of phage infection, lysogeny, and bacterial growth may influence treatment efficacy. Along with the infection-specific considerations described in the following Section 4.3, these factors should be taken into account when developing PAS strategies.

### 4.3. Other Considerations

#### 4.3.1. Endogenous Prophage Landscape

Several antibiotics including ciprofloxacin, which synergizes with certain temperate phages in vitro [58], have also been shown to induce endogenous prophages from gut bacteria [138]. While the potential for off-target induction events in vivo is not inherently surprising, other work suggests that off-target induction of certain virulence-regulating prophages could potentially exacerbate infections [139]. Specifically, reports suggest that clinical isolates of *S. aureus* are often lysogenized by *hlb*-converting prophages, which integrate into, and disrupt the expression of the virulence factor, β-hemolysin [139]. Treatment of *S. aureus* isolates from cystic fibrosis (CF) patients with subinhibitory trimethoprim or ciprofloxacin restored β-hemolysin production, indicating that gene-disrupting *hlb*-converting phages had been induced [139]. Researchers also reported phage excision due to subinhibitory ciprofloxacin exposure resulted in increased expression of the phage-encoded staphylokinase gene, *sak*, which is known to support bacterial virulence through several processes including plasminogen activation and deactivation of host-produced antimicrobial peptides [140]. Notably, induction of another *hlb*-converting prophage, ϕSa3mw with SOS-inducing agents mitomycin C and H_2_O_2_ in a different study did not result in viral particle production, suggesting that excision-mediated virulence regulation for this group of phages can occur without causing cell lysis [141]. These findings indicate that drug-induced prophage excision may increase bacterial virulence without producing canonical antibacterial effects. Furthermore, in *Listeria monocytogenes* prophage excision has been shown to protect cells from phagocytosis by macrophages [142]. Thus, off-target induction of endogenous prophages upon application of SOS-inducing antibiotics could potentially increase the virulence of certain pathogens and requires situational consideration.

#### 4.3.2. Interactions with Innate Immune System

Despite the resolute inability of bacteriophages to infect human cells, a growing body of literature suggests that certain phages induce human immune responses [143]. For phage therapy, the consequences of phage-driven immune activation are diverse, and range from maladaptive effects such as virion inactivation and reduced pathogen control, to synergistic antibacterial effects [144,145,146,147]. While these maladaptive effects may threaten PAS efficacy in an immune-competent host, certain phage-based treatments could be more effective in the presence of immune factors. For instance, Roach et al. found that *P. aeruginosa* phage PAK_P1 effectively cleared *P. aeruginosa*-induced acute pneumonia in WT mice, but not in MyD88-deficient, lymphocyte deficient, neutrophil-deficient mice [147]. Further in silico modeling predicted that immune system components are required to fully clear phage-sensitive *P. aeruginosa*, as well as phage-resistant subpopulations arising during treatment [147]. Despite these findings, clear examples of immune system contributions to PAS remain limited. In silico studies modeling PAS in immune-competent and immune-deficient hosts have predicted that the innate immune system supports bacterial clearance by *P. aeruginosa* phages and ciprofloxacin, and may represent a key determinant of treatment success in vivo [148]. Other studies have found that human serum lysozyme and human serum albumin synergize with the purified phage lysin, exebacase [149]. Notably, the concentration of exebacase required for synergy with daptomycin decreased from a previously reported value of 4 ug/mL in nutrient-rich lab media [63] to 0.025 ug/mL in human serum, which posits that serum factors may enhance PAS against *S. aureus* [149]. Similarly, the synergistic killing of *A. baumanii* by depolymerase DPo71 and colistin required supplementation of at least 5% human serum, suggesting that innate immunity factors present in serum contribute to treatment efficacy in that system [64]. Given these findings and the established immunogenic potential of phages [143], the cooperative or antagonistic effects of human immune factors on phage–antibiotic combinations warrant additional study.

#### 4.3.3. Polymicrobial Infections

Host-specific differences could prevent the application of findings derived from single-pathogen studies to PAS in polymicrobial infection settings, where interspecies competition may influence drug susceptibility [150]. This phenomenon has been observed for antibiotic combination treatments of gentamicin and ceftazidime, which reduce viable cell counts of *P. aeruginosa*, but demonstrate antagonistic activity when applied to a polymicrobial community of *P. aeruginosa*, *S. aureus*, *E. faecalis*, and *A. baumannii* [150]. To date, research surrounding PAS in polymicrobial systems is limited [117,151,152]. However, in one study, researchers noted that phage–antibiotic treatment of *P. aeruginosa* in dual-species biofilms with phage EPA1 required significant alteration of antibiotic concentration in comparison to single-species experiments [152], highlighting a need for more research considering mixed pathogen populations. In addition to impacting antimicrobial dosage, the interspecies competition in polymicrobial cultures may promote the evolution of CRISPR-based phage immunity [153], which could potentially interfere with phage activity in PAS treatments. Thus, as other reviews have also mentioned [52], the presence of multiple pathogens in a single infection could significantly impact treatment outcomes and represent an important area for future investigation.

## 5. Conclusions

### 5.1. Summary of Insights

Phage–antibiotic synergy represents a sophisticated strategy for combatting MDR pathogens that is driven by a wide range of molecular and evolutionary mechanisms. However, antagonistic interactions can occur, and are a major consideration toward developing phage–antibiotic strategies [105,106,108]. Previously, Gu Lui et al. suggested that the mechanism of antibiotic activity, as it relates to phage replication, the concentration of each antimicrobial, and the specific environmental conditions under which PAS is tested are important considerations when developing PAS strategies [122]. Building upon these insights, the known mechanisms of synergy and antagonism reviewed here highlight the idea that formulation of phage–antibiotic pairs requires integrated consideration of (I) antibiotic mechanisms of action, (II) phage lifestyle and replication requirements, (III) phage receptor identity, and (IV) host antibiotic resistance mechanisms. During the development of PAS strategies against specific pathogens, it is possible that the integration of these considerations may point researchers toward effective phage–host combinations. However, substantial gaps toward understanding the basis of phage–antibiotic interactions remain obstacles toward strategic treatment development.

### 5.2. Research Gaps and Future Directions

Clear-cut patterns of PAS across phage, antibiotic, or pathogen types are difficult to derive because the current body of literature describing the underlying mechanisms of phage–antibiotic interactions across diverse systems remains limited [45,50]. Similarly, recent works suggest that a range of mechanisms underly phage–antibiotic antagonism (Section 3), but elucidation of these mechanisms across systems is limited and antagonism is rarely investigated under infection-relevant conditions. Indeed, mechanistic investigations of the antagonistic or synergistic interactions of phage–antibiotic combinations in treatment-relevant settings such as in polymicrobial communities, and in the presence of human immune factors, would support the design of effective PAS strategies. However, cross-study comparisons are difficult to draw due to the variability in the methods and parameters used to identify PAS. Thus, the establishment of a clear framework for developing phage–antibiotic treatments requires the standardization of in vitro testing methods [125,126]. Given the dynamic nature of phage–antibiotic interactions across time, concentration, and testing conditions as described in Section 3.2.3, testing methods that collect a range of dynamic and static data outputs, such as the synograph [122], may be especially useful for mechanistic investigations of PAS. Future work aimed at standardizing in vitro testing methods may build upon these advances and seek to extend PAS investigations across a wider range of systems and conditions.

## Data Availability

No new data were created in this study. Data sharing is not applicable to this article.

## Abbreviations

The following abbreviations are used in this manuscript:

PAS	Phage–Antibiotic Synergy
AMR	Antimicrobial Resistance
LPS	Lipopolysaccharide
SOS	“Save-Our-Souls”
